# Supplementary material for: Lysosomal exocytosis releases pathogenic α-synuclein species from neurons in synucleinopathy models
Source: Nat Commun. 2022 Aug 22;13:4918. doi: 10.1038/s41467-022-32625-1 (PMC9395532; doi:10.1038/s41467-022-32625-1)
Supplement: Supplementary file 1 — Supplementary Information [file 41467_2022_32625_MOESM1_ESM.pdf]

SUPPLEMENTARY INFORMATION

Lysosomal Exocytosis Releases Pathogenic α-Synuclein Species from Neurons

Ying Xue Xie, Nima N. Naseri, Jasmine Fels, Parinati Kharel, Yoonmi Na, Diane Lane, Jacqueline Burré, Manu Sharma

CONTENTS

Abbreviations List

Supplementary Figures S1-S10

Supplementary Table 1

Supplementary References

ABBREVIATIONS LIST:

**2-APB**	2-Aminoethoxydiphenylborate
**αSyn**	α-Synuclein
**αSyn^{Amyl}**	α-Synuclein (aggregates) amyloid-type
**αSyn^{Fila}**	α-Synuclein (aggregates) filamentous
**Aggr**	Aggregate
**Amyl.**	Amyloid
**ANCL**	Adult-onset ceroid lipofuscinosis
**ANOVA**	Analysis of variation
**APEX2**	Ascorbate peroxidase enzyme derivative
**APV**	Amino-5-phosphonopentanoic acid
**ATP5G**	ATP synthase subunit 5g, mitochondrial
**BSA**	Bovine Serum Albumin
**Cath-D**	Cathepsin-D
**$Cath\text{-}L^{Mat}$**	Cathepsin-L; Mat.=mature
**CD81**	Cluster of Differentiation 81
**Citr. Synth.**	Citrate synthase
**CNQX**	6-Cyano-7-nitroquinoxaline-2,3-dione
**Coom. Blue**	Coomassie Blue
**CSF**	Cerebrospinal fluid
**CSPα**	Cysteine string protein-alpha
**Dan**	Dantrolene
**DAPI**	4′,6-diamidino-2-phenylindole
**DIV**	days in vitro
**DN**	dominant-negative
**E.R.**	Endoplasmic reticulum
**Early endo.**	Early endosome
**EEA1**	Early Endosome Antigen 1
**Excit. Syn.**	Excitatory Synapse
**Fila.**	Filament
**Frac**	Fraction
**Frz. Thaw**	Freeze thaw
**GAPDH**	Glyceraldehyde-3-phosphate dehydrogenase
**GFP**	green fluorescent protein
**GluN2B/NR2B**	Glutamate [NMDA] receptor subunit epsilon-2
**GOSR1**	Golgi SNAP receptor complex member 1
**GOSR2**	Golgi SNAP receptor complex member 2
**HA**	hemagglutinin
**HSP60**	Heat shock protein 60kDa
**IB**	Immunoblot
**Imm-Isol'd**	Immunoisolated
**Inhib. Syn.**	Inhibitory Synapse
**IP**	Immunoprecipitation
**K114**	Amyloid fibril-specific fluorescent dye: 4,4'-[(2-Bromo-1,4-phenylene)di-(1E)-2,1-ethenediyl]bisphenol
**KD**	knockdown
**LAMP1**	Lysosome-associated membrane glycoprotein 1
**LAMP2**	Lysosome-associated membrane glycoprotein 2

**LDH**	Lactate dehydrogenase
**Lys.**	Lysate
**Map2**	Microtubule-associated protein 2
**Med**	Media
**Mitoch**	Mitochondria
**Mo**	month
**Mono**	Monomer
**n.s.**	not significant
**Na/K-ATPase**	Sodium-potassium ATPase pump
**NeuN**	Neuronal Nuclei
**PD**	Parkinson’s disease
**Perox.**	Peroxisome
**Pex13**	Peroxisomal membrane protein 13
**Pex3**	Peroxisomal biogenesis protein 3
**PK**	Proteinase K
**PLA**	Proximity ligation assay
**Plasma memb.**	Plasma membrane
**pSer129**	phosphoSerine129
**Resc.**	Rescue
**SDS**	Sodium dodecyl sulfate
**Sec22b**	Vesicle trafficking protein homolog b
**Sec22c**	Vesicle trafficking protein homolog c
**Sec22L2**	Vesicle trafficking protein homolog a
**SNAP**	Synaptosomal-associated protein
**SNARE**	Soluble NSF Attachment Protein Receptor
**Syn. Ves.**	Synaptic Vesicle
**Synap1**	Synapsin-1
**Syngr-1**	Synaptogyrin-1
**Term**	Terminal
**TEV**	tobacco etch virus
**TGN38**	Trans-Golgi network integral membrane protein 2
**Tg-αSynA53T**	Transgenic α-Synuclein mouse with A53T mutation
**Tg^{x2}-αSynA53T**	homozygous transgenic α-Synuclein mouse with A53T mutation
**ThT**	Thioflavin-T
**TIM23**	Mitochondria Inner Membrane translocase subunit 23
**TMEM192**	Transmembrane protein 192
**Tot. lys.**	Total Lysate
**Trunc.**	Truncation
**TSG101**	Tumor susceptibility gene 101 protein
**TTX**	Tetrodotoxin
**Tuj1**	β-tubulin III
**TX100**	Triton X-100
**Uncon. Med**	Unconditioned Media
**VAMP**	Vesicle-associated membrane protein
**VTI1A**	Vesicle transport through interaction with T-SNAREs 1A
**VTI1B**	Vesicle transport through interaction with T-SNAREs 1B
**WPRE**	Woodchuck Hepatitis Virus Posttranscriptional Regulatory Element
**WT**	Wildtype

**Ykt6**	Synaptobrevin homolog
**YM-58483**	N-[4-[3,5-Bis(trifluoromethyl)-1H-pyrazol-1-yl]phenyl]-4-methyl-1,2,3-thiadiazole-5-carbox-amide

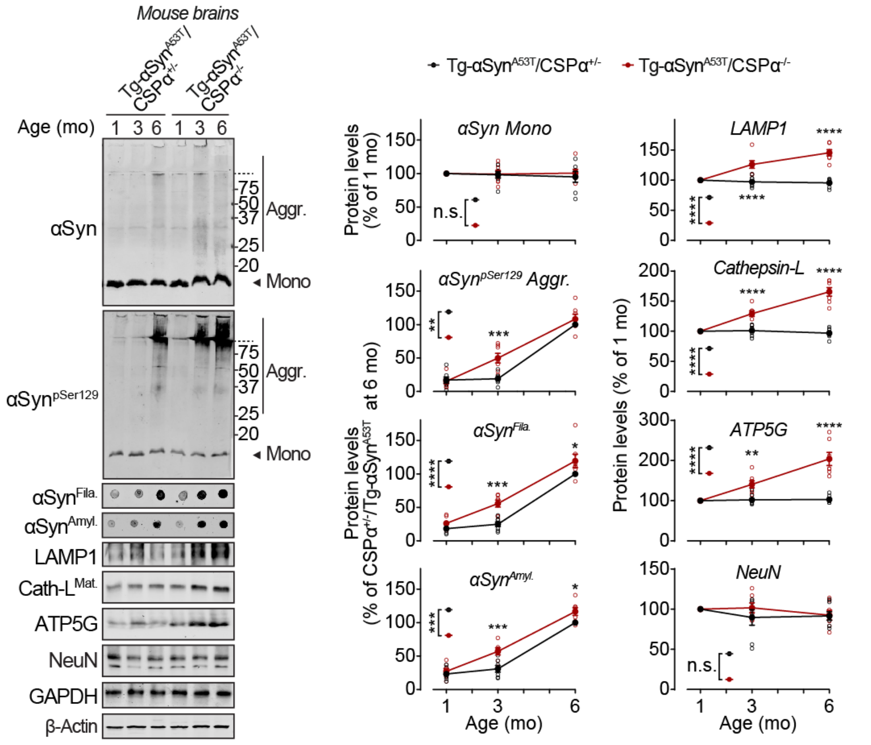

Supplementary Figure S1 | Accelerated accumulation of pathogenic αSyn aggregates and lysosomal proteins in Tg-αSyn^{A53T} mouse brains due to the loss of CSPα function.

Brains collected from Tg-αSyn^{A53T}/CSPα$^{+/-}$ and Tg-αSyn^{A53T}/CSPα$^{-/-}$ littermates at 1, 3 and 6 months of age were analyzed by quantitative immunoblotting for the following versions of αSyn: monomeric (αSyn Mono), phosphorylated at Ser129 (αSyn^{pSer129}), filamentous (αSyn^{Fila}), and amyloid-type (αSyn^{Amyl}); for lysosomal proteins LAMP1 and cathepsin-L (Cath-L^{Mat}); for ATP5G, which accumulates in lysosomal storage caused by loss of CSPα function; as well as for neuronal marker NeuN. These were all normalized to β-actin levels. Mono = monomer; Aggr. = aggregates. (n=7 mice at each age-point for each genotype). All data are shown as means ± SEM, where 'n' represents littermate mouse brains. n.s. = not significant; *P<0.05; **P<0.01; ***P<0.001; ****P<0.0001 by 2-way ANOVA with Bonferroni multiple comparisons post-test.

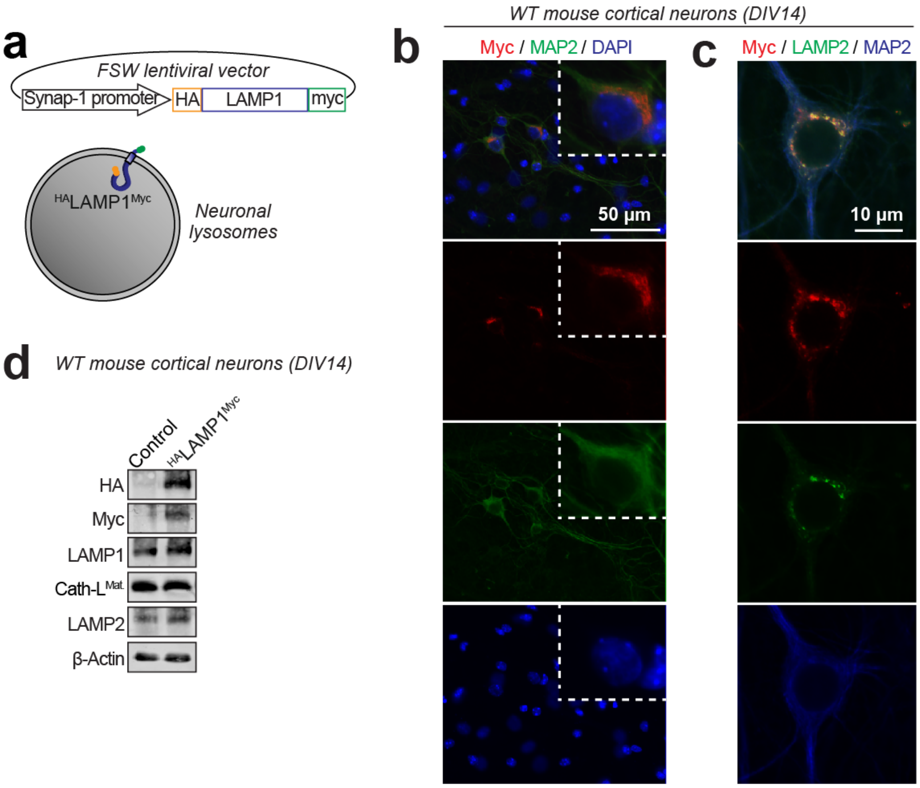

Supplementary Figure S2 | Neuronally expressed ^{HA}LAMP1^{Myc} lentiviral construct.

(**a-d**) ^{HA}LAMP1^{Myc} construct lentivirally expressed via neuron-specific synapsin-1 promoter shown as a schematic (**a**), localizes in neurons marked by MAP2, and not in surrounding glia seen in DAPI panel (**b**), and co-localizes with the late-endosomal/lysosomal protein LAMP2 in primary neuron cultures (**c**). Expression of full-length protein ^{HA}LAMP1^{Myc} is confirmed by immunoblotting for its epitope tags: N-terminal HA tag and C-terminal myc tag. (**b-d**) Representative images from n=3 independent cortical cultures plus lentiviral transduction experiments. *Note:* Further characterization of Tg-^{HA}LAMP1^{Myc} expressing mice based on this construct is included in **Fig. 2** and in **Supplementary Fig. S3**.

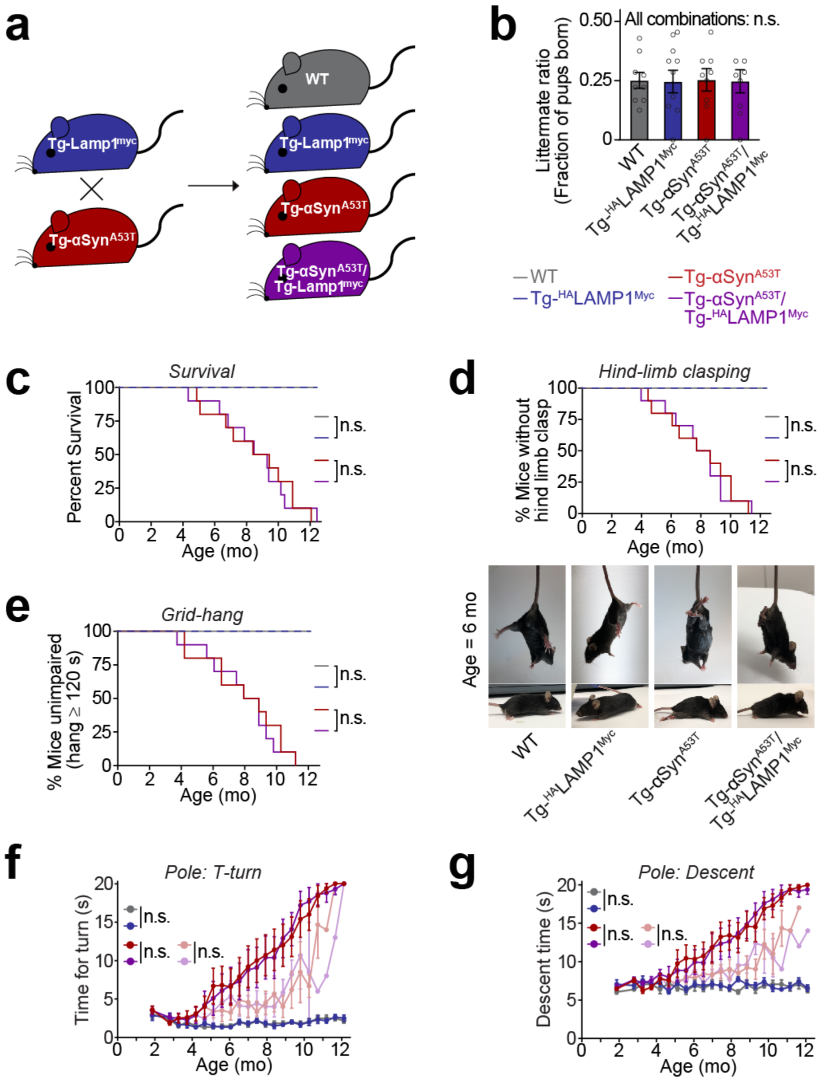

Supplementary Figure S3 | In Tg-αSyn^{A53T}/Tg-^{HA}LAMP1^{Myc} double-transgenic mice, the neuromuscular impairments driven by αSyn^{A53T} transgene remain unaffected by the ^{HA}LAMP1^{Myc} transgene.

(**a**) Tg-αSyn^{A53T} mice were crossed to Tg-^{HA}LAMP1^{Myc} mice to generate the Tg-αSyn^{A53T}/Tg-^{HA}LAMP1^{Myc} progeny. (**b**) Equal numbers of pups with the four resultant genotypes (WT, Tg-^{HA}LAMP1^{Myc}, Tg-αSyn^{A53T}, and Tg-αSyn^{A53T}/Tg-^{HA}LAMP1^{Myc}) were born from crossing Tg-αSyn^{A53T} and Tg-^{HA}LAMP1^{Myc} parents (n=9 litters). Tg-αSyn^{A53T}/Tg-^{HA}LAMP1^{Myc} and Tg-αSyn^{A53T} mice were indistinguishable in (**c**) lifespan, as well as in the onset and course of neuromuscular deterioration (**d-g**): Signs of progressive neuromuscular debility preceded death, as measured by (**d**) onset of hind-limb clasping, (**e**) grid-hang (limb strength), (**f**) T-turn on pole-test (bradykinesia), and (**g**) descent on pole test (motor coordination) (n=6 mice per group for WT and Tg-^{HA}LAMP1^{Myc}, n=10 mice per group for Tg-αSyn^{A53T} and Tg-αSyn^{A53T}/Tg-^{HA}LAMP1^{Myc}). (**f-g**) Tg-αSyn^{A53T} and Tg-αSyn^{A53T}/Tg-^{HA}LAMP1^{Myc} mice that died were scored as 20 s (maximum time allowed for measurement) for the rest of the trial – not as an actual measurement, but as a place holder (to avoid seeming improvement of the cohort after each death). The same data are shown in the lighter shaded graphs without the 20 s placeholder, as dead animals are eliminated from the cohort over time. WT and Tg-^{HA}LAMP1^{Myc} mice showed no impairments in these measurements (n=5 mice per group). (**b and f-g**) Data represent means ± SEM. (**b**) n.s. = not significant, by RM 1-way ANOVA, (**c-e**) Log-rank (Mantel-Cox) test for comparisons of Kaplan-Meier curves, (**f-g**) RM 2-way ANOVA for genotype

comparisons in dark shaded graphs and mixed-effects analysis for Tg-αSyn^{A53T} and Tg-αSyn^{A53T}/Tg-^{HA}LAMP1^{Myc} comparisons (lighter shaded graphs) due to mouse mortality.

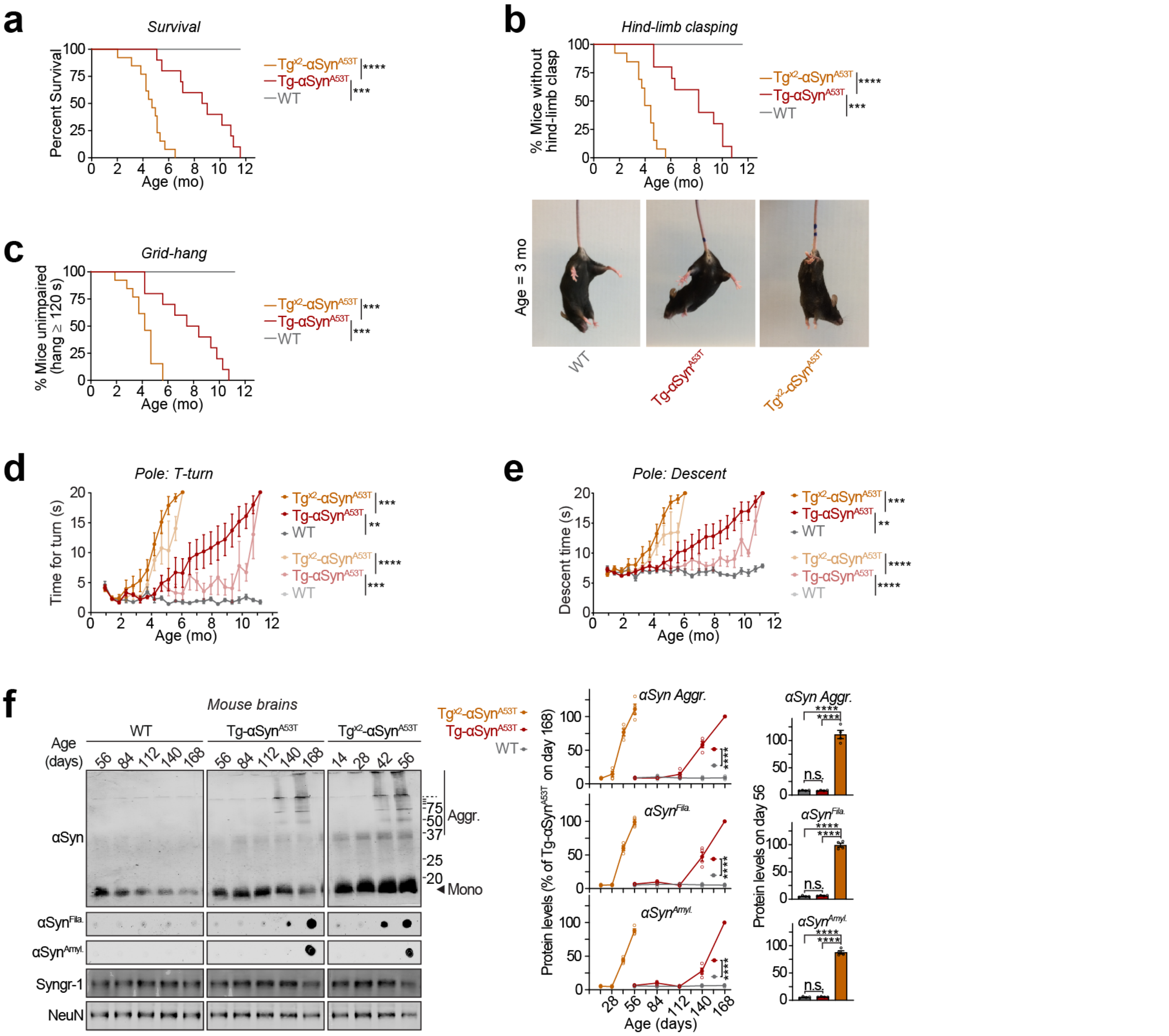

Supplementary Figure S4 | Characterization of the homozygous Tg^{x2}-αSyn^{A53T} mice.

(**a**) Doubling the dose of αSyn^{A53T} in Tg^{x2}-αSyn^{A53T} mice resulted in accelerated mortality, nearly halving the lifespan of Tg-αSyn^{A53T} mice. This is accompanied by accelerated onset of neuromuscular impairment in Tg^{x2}-αSyn^{A53T} mice, as measured by (**b**) onset of hind-limb clasping, (**c**) onset of grid-hang impairment (limb strength). (**d**) T-turn on pole-test (bradykinesia), and (**e**) descent on pole test (motor coordination). WT mice showed no impairments during the time course (n=10 Tg-αSyn^{A53T}; n=13 Tg^{x2}-αSyn^{A53T}; n=6 WT mice). (**d-e**) Tg-αSyn^{A53T} and Tg^{x2}-αSyn^{A53T} mice that died were scored as 20 s (maximum time allowed for measurement) for the rest of the trial – not as an actual measurement, but as a place holder (to avoid seeming improvement of the cohort after each death). The same data are shown in the lighter shaded graphs without the 20 s placeholder, as dead animals are eliminated from the cohort over time. (**f**) In addition to increased levels of αSyn monomer, αSyn aggregates are detected in Tg^{x2}-αSyn^{A53T} brains much earlier than in Tg-αSyn^{A53T} brains. Both, time course and bar-graph are normalized to NeuN levels (n=4 mice per genotype). (**a-c**) ***P<0.001 and ****P<0.0001 by

Log-rank (Mantel-Cox) test. (**d**-**f**) Data represent means ± SEM, $^{**}P<0.01$; $^{***}P<0.001$; $^{****}P<0.0001$; (**d**-**e**) RM 2-way ANOVA (dark shaded graphs) and mixed-effects analysis due to mouse mortality (lighter shaded graphs); analysis between Tg^{x2}-αSyn^{A53T} and Tg-αSyn^{A53T} is up to 6 mo; (**f**) time-course comparison between WT and Tg-αSyn^{A53T}: 2-way ANOVA, bar graph: 1-way ANOVA with Bonferroni multiple comparisons post-test.

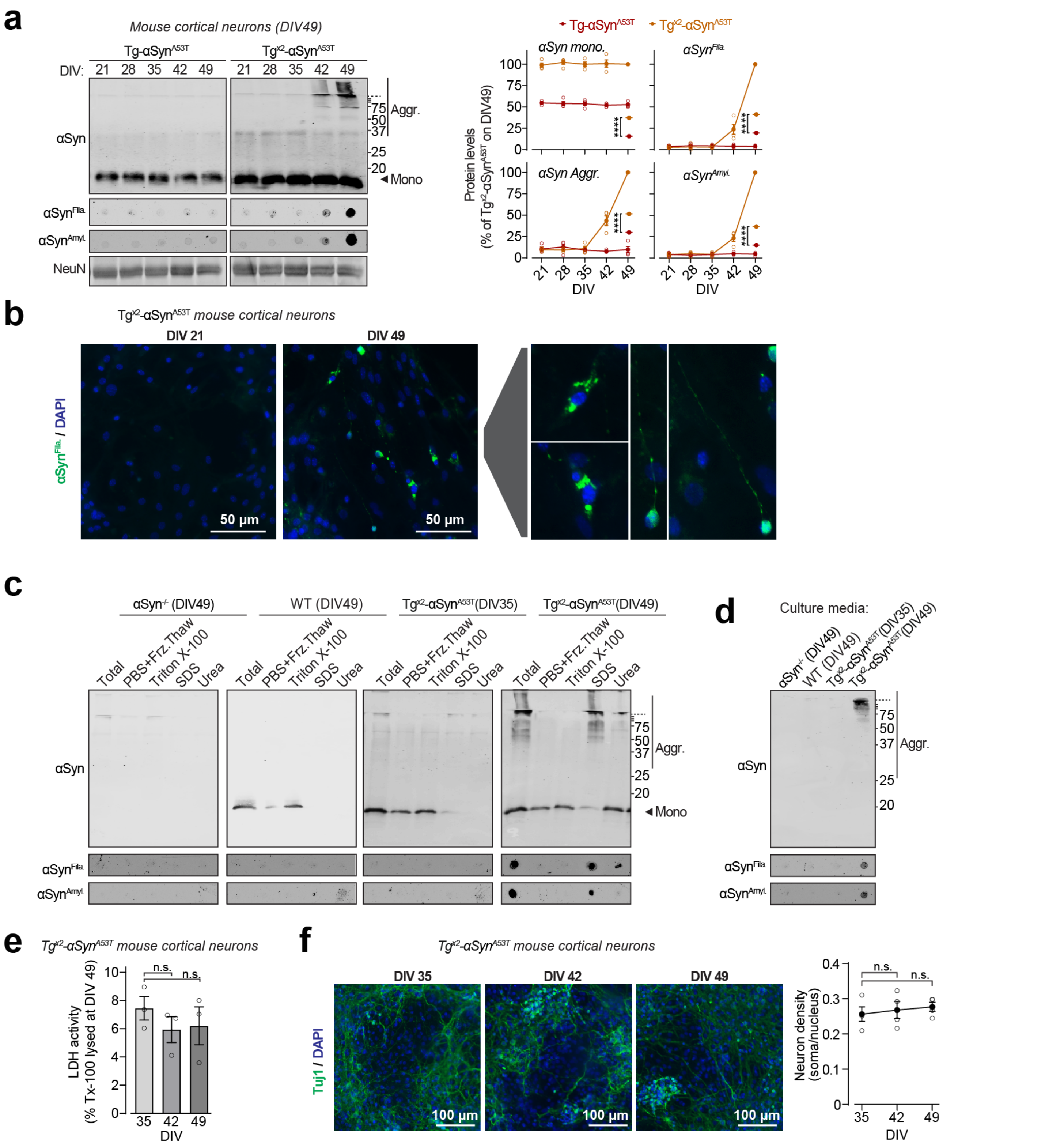

Supplementary Figure S5 | Characterization of homozygous Tg^{x2}-αSyn^{A53T} primary neurons.

(**a**) Cortical primary neurons from Tg^{x2}-αSyn^{A53T} mice, but not from Tg-αSyn^{A53T} mice accumulate pathogenic αSyn species by DIV42, detected via immunoblots using the indicated antibodies (αSyn$^{Fila.}$ = filamentous αSyn; αSyn$^{Amyl.}$ = Amyloid-type αSyn). Levels of αSyn species are normalized to NeuN levels (n=4). (**b**) The aggregates were detectable *in situ* at DIV49, but not at DIV21, via antibodies against filamentous αSyn (αSyn$^{Fila.}$). Right panels: magnified images showing aggregates of various sizes in cell bodies, as well as within neurites (representative from n=3). (**c**) Cortical neuron cultures

from αSyn knockout (αSyn$^{-/-}$; DIV49), wild type (WT; DIV49), and Tg^{x2}-αSyn^{A53T} (DIV35 and 49) were serial-extracted by freeze-thaw, Triton X-100 (1%), SDS (1%), and urea (8M) and immunoblotted for the indicated species of αSyn (representative from n = 3). (**d**) Media from the cultures shown in panel (**c**) were collected following 48 h conditioning prior to harvesting, and immunoblotted for indicated versions of αSyn (representative from n=3). (**e**) Media from Tg^{x2}-αSyn^{A53T} neurons conditioned for 48 h prior to DIV35, 42 and 49 was analyzed for extracellular lactate dehydrogenase (LDH) as a measure of cell death, and is shown as % of LDH activity in Triton X-100 lysate of the DIV49 culture (n=3). (**f**) Tg^{x2}-αSyn^{A53T} neurons were immunolabeled with Tuj1 at DIV35, 42 and 49 and neuron survival was measured as neuron density = neuron cell bodies/total nuclei (n=4). (**a** and **e-f**) Data represent means ± SEM, where n = independent primary culture performed from separate batch of litter(s). n.s. = not significant; **P<0.01; ***P<0.001; ****P<0.0001; (**a**) 2-way ANOVA; (**e**-**f**) 1-way ANOVA with Dunnett's multiple comparisons test.

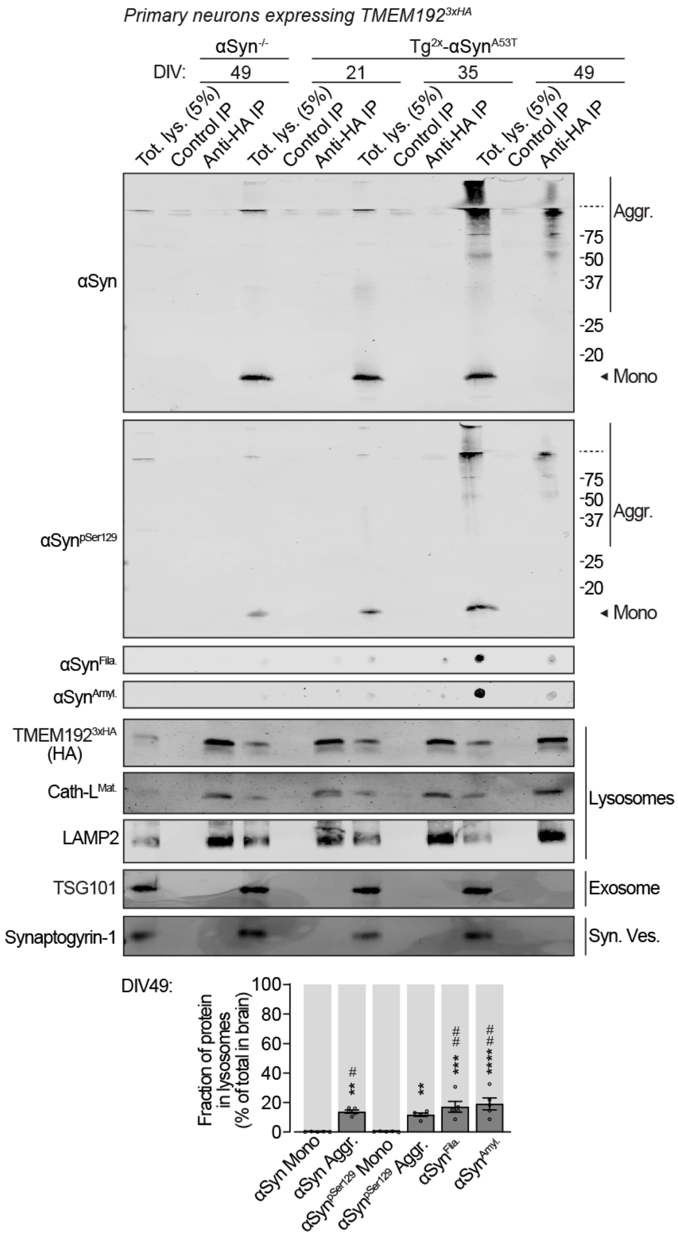

Supplementary Figure S6 | Pathogenic αSyn species accumulate within lysosomes in primary neurons.

Lysosomes were immunoisolated from either αSyn$^{-/-}$ neurons at 49 days *in vitro* (DIV49) or from Tg^{x2}-αSyn^{A53T} neurons at indicated DIVs, via lentiviral expression of lysosomal protein TMEM192 tagged with 3xHA on the cytosolic C-terminus [1,2]. Total cell lysate (5%) and immunoisolates (Control = no Ig IP) were immunoblotted for levels of αSyn, αSyn^{pSer129}, α-Syn-^{Fila}, and αSyn^{Amyl} (Mono = monomer, Aggr = aggregates), as well as markers of lysosomes (LAMP2 and mature cathepsin-L=Cath-L$^{Mat.}$), exosomes (TSG101), and synaptic vesicles (Syn. Ves.; synaptogyrin-1). The fraction of these αSyn species residing within the lysosomes at DIV49 is back-calculated from the fraction of each αSyn species immunocaptured (αSyn species immunocaptured/total input), normalized to the fraction of late-endosomes/lysosomes captured – indicated by the fraction of TMEM192^{3xHA} captured (HA blot immunocaptured/total input). Immunoblots against markers of lysosomes are also shown. No αSyn protein (monomer or aggregate) was detected in αSyn$^{-/-}$ and no αSyn aggregates were present in Tg^{x2}-αSyn^{A53T} at DIV 21 and 35, in either total lysates or lysosomes, Thus αSyn from these conditions could not be accurately quantified, and only quantification for DIV49 in Tg^{x2}-αSyn^{A53T} is shown (n=5). Data represent means ± SEM, where each ‘n’ corresponds to independent culture and immunoisolation. **$P<0.01$, ***$P<0.001$, ****$P<0.0001$ by 1-way ANOVA

with Dunnett multiple-comparison correction; and $^{\#}P<0.05$, $^{\#\#}P<0.01$ by non-parametric Kruskal-Wallis test with Dunn's multiple-comparison adjustment; all tests comparing levels of each αSyn aggregate-type to αSyn monomer.

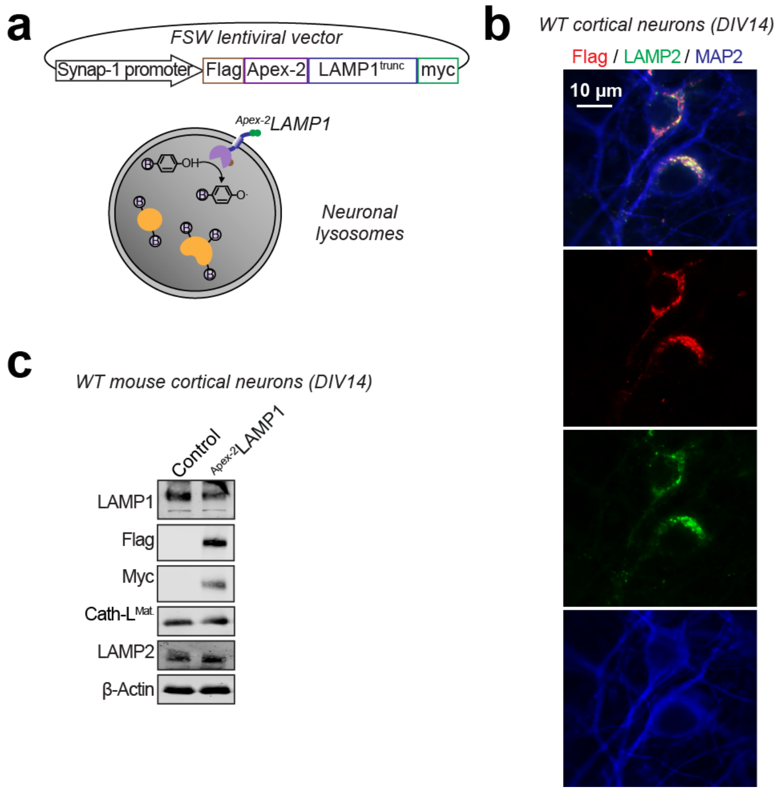

Supplementary Figure S7 | Generation of $^{Apex\text{-}2}$LAMP1 chimeric protein for labeling lysosomal luminal proteins in primary neurons.

(**a**) Apex-2 was fused to the N-terminus of truncated LAMP1 and expressed via neuron-specific synapsin-1 promoter, allowing it to biotinylate late-endosomal/lysosomal luminal proteins. (**b**) Lentivirally expressed $^{Apex\text{-}2}$LAMP1 (infected on DIV7, immunostained on DIV14) co-localizes with late-endosomal/lysosomal protein LAMP2 in primary neurons. Neuronal soma and dendrites are marked by MAP2. (**c**) Expression of $^{Apex\text{-}2}$LAMP1 in primary neurons is confirmed by immunoblotting for its epitope tags: N-terminal FLAG and C-terminal myc. (**b-c**) Representative images from n=3 independent cortical cultures plus lentiviral transduction experiments.

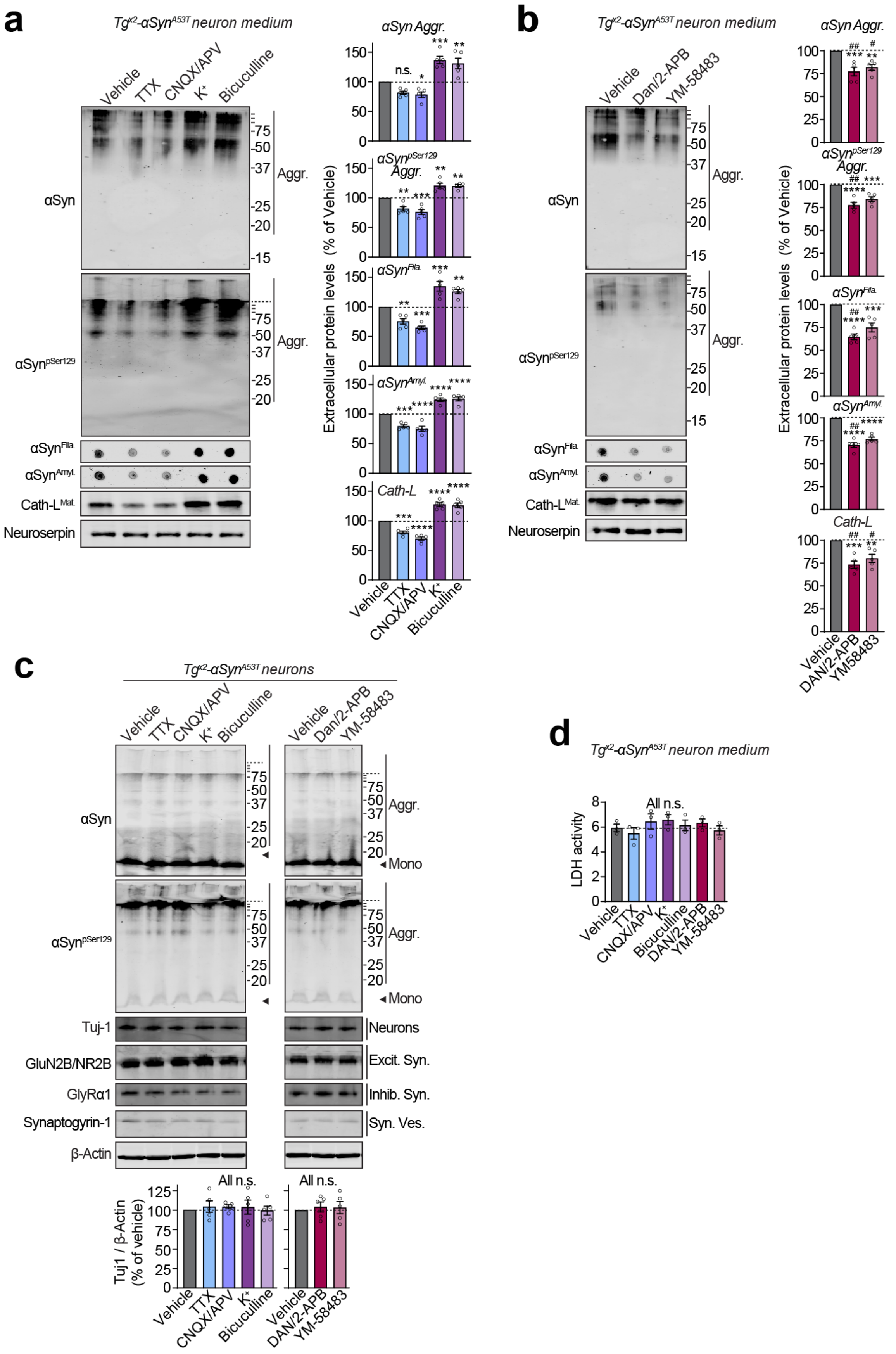
a
Tg^x2-αSyn^A53T neuron medium
Vehicle
TTX
CNQX/APV
K⁺
Bicuculline
αSyn
αSyn^pSer129
αSyn^Fila.
αSyn^Amyl.
Cath-L^Mat.
Neuroserpin
Aggr.
αSyn Aggr.
αSyn^pSer129 Aggr.
αSyn^Fila.
αSyn^Amyl.
Cath-L
Extracellular protein levels (% of Vehicle)
n.s.
b
Tg^x2-αSyn^A53T neuron medium
Vehicle
Dan/2-APB
YM-58483
DAN/2-APB
YM58483
c
Tg^x2-αSyn^A53T neurons
Mono
Tuj-1
GluN2B/NR2B
GlyRα1
Synaptogyrin-1
β-Actin
Neurons
Excit. Syn.
Inhib. Syn.
Syn. Ves.
All n.s.
Tuj1 / β-Actin (% of vehicle)
d
Tg^x2-αSyn^A53T neuron medium
LDH activity
All n.s.

Supplementary Figure S8 | Neuronal activity and cytosolic calcium-dependence of lysosomal and αSyn aggregate exocytosis.

(**a-d**) Cortical primary neurons from Tg^{x2}-αSyn^{A53T} mice, were treated with indicated pharmacological agents at DIV48 or vehicle (0.1% DMSO) for 24 h, to manipulate neuronal activity (**a**) or to reduce cytosolic Ca^{2+} levels (**b**). Media were collected and analyzed by immunoblotting against the indicated species of αSyn, and cathepsin-L as an indicator of lysosomal exocytosis (n=5). (**c**) Cell lysates were also immunoblotted for neuronal and synaptic markers to control for neuron/synapse levels on DIV49. Tuj-1 levels were quantified as indicator of neuron survival and normalized to β-actin levels (n=5). (**d**) To test further for cell death, LDH activity in the medium was quantified in DIV49 media (n=3). All data represent means ± SEM. Each 'n' is an independent primary culture and its pharmacological treatment. n.s. = not significant; *P<0.05; **P<0.01; ***P<0.001; ****P<0.0001 by 1-way ANOVA with Dunnett's multiple-comparison correction, and $^{\#}P<0.05$; $^{\#\#}P<0.01$ by non-parametric Kruskal-Wallis test with Dunn's multiple-comparison adjustment.

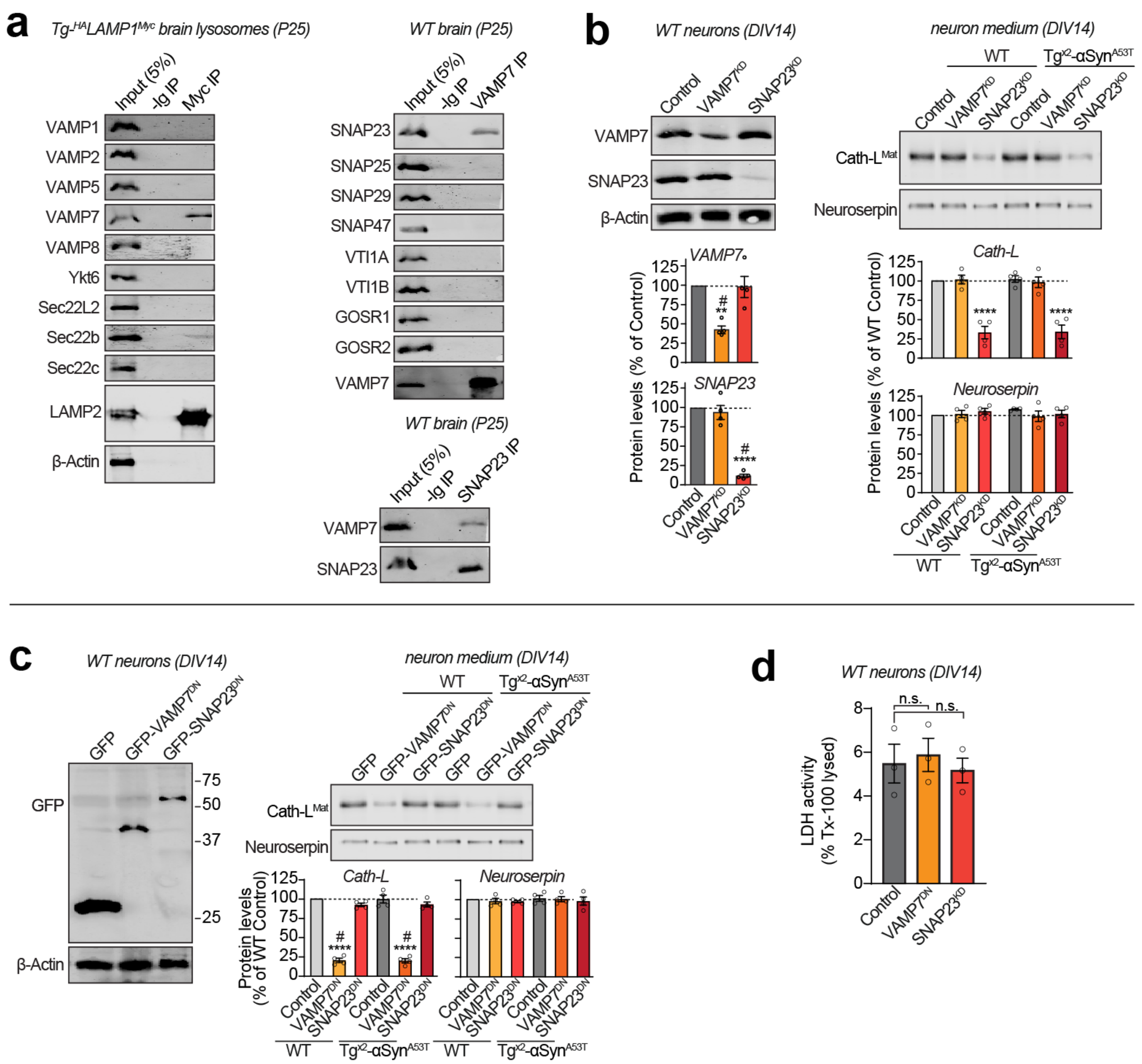

Supplementary Figure S9 | SNARE-dependence of lysosomal exocytosis.

(**a**) From Tg-^{HA}LAMP1^{Myc} mouse brains, we immunoisolated lysosomes (as in Fig. 3), followed by immunoblotting for the indicated v-SNAREs (left panel). From wild type mouse brains, we immunoprecipitated (IP) VAMP7 and immunoblotted for the indicated co-IP'd Qb motif containing t-SNAREs (right top panel). We then performed the inverse IP, where SNAP23 was IP'd and VAMP7 co-IP was tested by immunoblotting (right bottom panel) (representative of n=3). (**b**) In WT neurons, shRNA knockdown constructs VAMP7^{KD} and SNAP23^{KD} were lentivirally expressed (Control = virus without shRNA), followed by quantitative immunoblotting of VAMP7 and SNAP23 levels (n=4). Secreted levels of lysosome luminal protein cathepsin-L (Cath-L^{Mat}) and the non-lysosomal secreted protein neuroserpin were quantified from the medium of WT and Tg^{x2}-αSyn^{A53T} neurons, and normalized to total protein in media quantified by BCA assay (n=4). (**c**) In WT neurons, dominant-negative constructs GFP-VAMP7^{DN} and GFP-SNAP23^{DN} were lentivirally expressed (Control = GFP), followed by immunoblotting for GFP (representative of n=4). Secreted levels of lysosome luminal protein cathepsin-L (Cath-L^{Mat}) and the non-lysosomal secreted protein neuroserpin were quantified from the medium of WT and Tg^{x2}-

αSyn^{A53T} neurons subjected to the $VAMP7^{DN}$ and $SNAP23^{DN}$ (control = GFP alone) and normalized to total protein in medium quantified by BCA assay (n=4). (**d**) Media from WT neurons conditioned for 48 h prior to DIV14 was analyzed for extracellular lactate dehydrogenase (LDH) as a measure of cell-death and is shown as % of LDH activity in Triton X-100 lysate (n=3). All data represent means ± SEM. Each ‘n’ is an independent immunoisolation/immunoprecipitation in (**a**) and an independently infected neuron culture in (**b-c**), and independent cultures in (**d**). In (**b-c**) $^{**}P<0.01$; $^{****}P<0.0001$ by 1-way ANOVA with Dunnett’s multiple-comparison correction; and $^{\#}P<0.05$ by non-parametric Kruskal-Wallis test with Dunn’s multiple-comparison adjustment. In (**d**) n.s. = not significant by 1-way ANOVA with Dunnett’s multiple comparisons test.

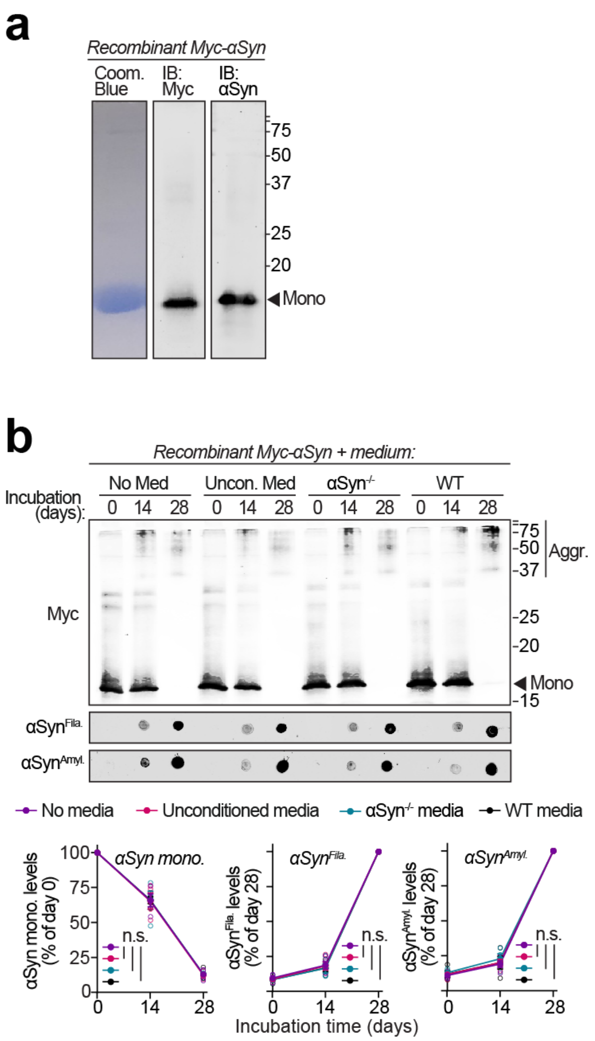

Supplementary Figure S10 | Effect of primary neuron media on aggregation of recombinant myc-αSyn.
(**a**) Purified recombinant myc-αSyn protein separated by SDS-PAGE, followed by Coomassie brilliant blue staining, as well as immunoblotting against myc and αSyn. (**b**) Recombinant purified myc-tagged αSyn (myc-αSyn) was incubated at 37°C in presence of no medium (PBS), unconditioned medium, or extracellular medium from mouse cortical neuron cultures collected over 48 h (DIV 47-49) generated either from αSyn knockout (αSyn$^{-/-}$) mice, or from wild type (WT) mice. Aggregation of myc-αSyn was analyzed at the indicated days of incubation by quantitative immunoblotting for the myc epitope-tag, filamentous myc-αSyn aggregates (αSyn^{Fila}), or amyloid-type myc-αSyn aggregates (αSyn^{Amyl}) using A11 antibody (n=4). (**a**) Representative images from n=4 independent αSyn purifications and immunoblots; (**b**) all data represent means ± SEM, where each ‘n’ is an independent media collection and aggregation experiment. n.s. = not significant by 2-way ANOVA with Bonferroni multiple comparisons post-test compared to no media.

Supplementary Table 1. Figure-by-figure ANOVA details.

Fig.	Dataset	Statistical test	Multiple Compar. test	ANOVA p-value	Multiple comparison p-value
2b	Male: WT v. Tg-^{HA}Lamp1^{Myc}	RM 2-way ANOVA (each mouse matched over age)		**main effect genotype: F (1, 8) = 0.02413, P=0.8804**; main effect age: F (10, 80) = 2277, P<0.0001; interaction: F (10, 80) = 0.5839, P=0.8225; mouse: F (8, 80) = 51.74, P<0.0001	
2b	Female: WT v. Tg-^{HA}Lamp1^{Myc}	RM 2-way ANOVA (each mouse matched over age)		**main effect genotype: F (1, 8) = 0.02490, P=0.8785**; main effect age: F (10, 80) = 883.7, P<0.0001; interaction: F (10, 80) = 0.2771, P=0.9846; mouse: F (8, 80) = 46.90, P<0.0001	
3a		1-way ANOVA	Dunnett	F (4, 35) = 97.95, P<0.0001	compared to monomer: Aggr: P<0.0001, pSer129Aggr: P<0.0001, Fila: P<0.0001, Amyl: P<0.0001
3a		Kruskal-Wallis	Dunn	<0.0001	compared to monomer: Aggr: P=0.2764, pSer129Aggr: P=0.0885, Fila: P<0.0001, Amyl: P<0.0001
3c	αSyn Aggr.	2-way ANOVA	Bonferroni	**main effect TX100: F (1, 30) = 223.9, P<0.0001**; main effect Proteinase K concentration: F (4, 30) = 65.25, P<0.0001; interaction: F (4, 30) = 29.89, P<0.0001	comparison of -/+ TX100 at each proteinase K concentration: at no proteinase K: P>0.9999; at 0.1ug/mL Proteinase K: P=0.0841; at 1ug/mL Proteinase K: P<0.0001; at 10ug/mL Proteinase K: P<0.0001; at 100ug/mL Proteinase K: P<0.0001
3c	αSyn pSer129 Aggr.	2-way ANOVA	Bonferroni	**main effect TX100: F (1, 30) = 225, P<0.0001**; main effect Proteinase K concentration: F (4, 30) = 92.48, P<0.0001; interaction: F (4, 30) = 34.09, P<0.0001	comparison of -/+ TX100 at each proteinase K concentration: at no proteinase K: P>0.9999; at 0.1ug/mL Proteinase K: P=0.3922; at 1ug/mL Proteinase K: P<0.0001; at 10ug/mL Proteinase K: P<0.0001; at 100ug/mL Proteinase K: P<0.0001
3c	αSyn Filament	2-way ANOVA	Bonferroni	**main effect TX100: F (1, 30) = 327.8, P<0.0001**; main effect Proteinase K concentration: F (4, 30) = 104.4, P<0.0001; interaction: F (4, 30) = 50.78, P<0.0001	comparison of -/+ TX100 at each proteinase K concentration: at no proteinase K: P>0.9999; at 0.1ug/mL Proteinase K: P=0.0566; at 1ug/mL Proteinase K: P<0.0001; at 10ug/mL Proteinase K: P<0.0001; at 100ug/mL Proteinase K: P<0.0001
3c	αSyn Amyloid	2-way ANOVA	Bonferroni	**main effect TX100: F (1, 30) = 397.3, P<0.0001**; main effect Proteinase K concentration: F (4, 30) = 129.6, P<0.0001; interaction: F (4, 30) = 62.79, P<0.0001	comparison of -/+ TX100 at each proteinase K concentration: at no proteinase K: P>0.9999; at 0.1ug/mL Proteinase K: P=0.068; at 1ug/mL Proteinase K: P<0.0001; at 10ug/mL Proteinase K: P<0.0001; at 100ug/mL Proteinase K: P<0.0001
3c	Cathepsin-L	2-way ANOVA	Bonferroni	**main effect TX100: F (1, 30) = 399.1, P<0.0001**; main effect Proteinase K concentration: F (4, 30) = 130.2, P<0.0001; interaction: F (4, 30) = 50.96, P<0.0001	comparison of -/+ TX100 at each proteinase K concentration: at no proteinase K: P>0.9999; at 0.1ug/mL Proteinase K: P=0.0023; at 1ug/mL Proteinase K: P<0.0001; at 10ug/mL Proteinase K: P<0.0001; at

					100ug/mL Proteinase K: P<0.0001
3c	ATP6V1B2	2-way ANOVA	Bonferroni	**main effect TX100: F (1, 30) = 0.5488, P=0.4646**; main effect Proteinase K concentration: F (4, 30) = 370.8, P<0.0001; interaction: F (4, 30) = 0.07105, P=0.9903	
3c	ATP6V1E2	2-way ANOVA	Bonferroni	**main effect TX100: F (1, 30) = 0.4414, P=0.5115**; main effect Proteinase K concentration: F (4, 30) = 588.1, P<0.0001; interaction: F (4, 30) = 0.2588, P=0.902	
4a	αSyn pSer129 Aggr.	2-way ANOVA	Bonferroni	**main effect TX100: F (1, 24) = 0.2241, P=0.6402**; main effect Proteinase K concentration: F (3, 24) = 104.2, P<0.0001; interaction: F (3, 24) = 0.09744, P=0.9607	
4a	αSyn Filament	2-way ANOVA	Bonferroni	**main effect TX100: F (1, 32) = 0.01132, P=0.9159**; main effect Proteinase K concentration: F (3, 32) = 442.9, P<0.0001; interaction: F (3, 32) = 0.7584, P=0.5257	
4a	αSyn Amyloid	2-way ANOVA	Bonferroni	**main effect TX100: F (1, 32) = 0.7323, P=0.3985**; main effect Proteinase K concentration: F (3, 32) = 425.6, P<0.0001; interaction: F (3, 32) = 0.3522, P=0.7878	
4a	Cathepsin-L	2-way ANOVA	Bonferroni	**main effect TX100: F (1, 24) = 0.003606, P=0.9526**; main effect Proteinase K concentration: F (3, 24) = 381.7, P<0.0001; interaction: F (3, 24) = 0.5300, P=0.6660	
4a	Neuroserpin	2-way ANOVA	Bonferroni	**main effect TX100: F (1, 24) = 0.000, P>0.9999**; main effect Proteinase K concentration: F (3, 24) = 804.4, P<0.0001; interaction: F (3, 24) = 0.4955, P=0.6888	
4a	TSG101	2-way ANOVA	Bonferroni	**main effect TX100: F (1, 32) = 144.7, P<0.0001**; main effect Proteinase K concentration: F (3, 32) = 149.0, P<0.0001; interaction: F (3, 32) = 47.67, P<0.0001	comparison of -/+ TX100 at each proteinase K concentration: at no proteinase K: P>0.9999; at 1ug/mL Proteinase K: P>0.9999; at 10ug/mL Proteinase K: P<0.0001; at 100ug/mL Proteinase K: P<0.0001
4b	αSyn pSer129 Aggr.	2-way ANOVA	Bonferroni	**main effect TX100: F (1, 16) = 0.02762, P=0.8701**; main effect Proteinase K concentration: F (3, 16) = 80.17, P<0.0001; interaction: F (3, 16) = 0.03853, P=0.9895	
4b	αSyn Filament	2-way ANOVA	Bonferroni	**main effect TX100: F (1, 16) = 3.408, P=0.0835;** main effect Proteinase K concentration: F (3, 16) = 1788, P<0.0001; interaction: F (3, 16) = 1.278, P=0.3156	
4b	αSyn Amyloid	2-way ANOVA	Bonferroni	**main effect TX100: F (1, 16) = 3.008, P=0.1021**; main effect Proteinase K concentration: F (3, 16) = 2170, P<0.0001; interaction: F (3, 16) = 1.183, P=0.3475	
4b	Cathepsin-L	2-way ANOVA	Bonferroni	**main effect TX100: F (1, 16) = 0.008257, P=0.9287**; main effect Proteinase K concentration: F (3, 16) = 280.1, P<0.0001; interaction: F (3, 16) = 0.05229, P=0.9836	
4b	Neuroserpin	2-way ANOVA	Bonferroni	**main effect TX100: F (1, 16) = 1.313, P=0.2687**; main effect Proteinase K concentration: F (3, 16) = 750.6, P<0.0001; interaction: F (3, 16) = 0.8704, P=0.4768	

4b	TSG101	2-way ANOVA	Bonferroni	**main effect TX100: F (1, 16) = 71.12, P<0.0001**; main effect Proteinase K concentration: F (3, 16) = 127.9, P<0.0001; interaction: F (3, 16) = 19.83, P<0.0001	comparison of -/+ TX100 at each proteinase K concentration: at no proteinase K: P>0.9999; at 1ug/mL Proteinase K: P=0.0478; at 10ug/mL Proteinase K: P<0.0001; at 100ug/mL Proteinase K: P=0.0108
5b	αSyn pSer129 Aggr.	1-way ANOVA	Dunnett	F (3, 24) = 7.339, P=0.0012	compared to control: Vamp7DN: P=0.0020, Snap23KD: P=0.0082, Snap23KD+Rescue: P>0.9999
5b	αSyn Filament	1-way ANOVA	Dunnett	F (3, 24) = 11.07, P<0.0001	compared to control: Vamp7DN: P=0.0001, Snap23KD: P=0.0011, Snap23KD+Rescue: P=0.4888
5b	αSyn Amyloid	1-way ANOVA	Dunnett	F (3, 24) = 9.259, P=0.0003	compared to control: Vamp7DN: P=0.0005, Snap23KD: P=0.0013, Snap23KD+Rescue: P=0.3887
5b	Cathepsin-L	1-way ANOVA	Dunnett	F (3, 24) = 10.69, P=0.0001	compared to control: Vamp7DN: P=0.0002, Snap23KD: P=0.0004, Snap23KD+Rescue: P=0.1738
5b	αSyn pSer129 Aggr.	Kruskal-Wallis	Dunn	0.0042	compared to control: Vamp7DN: P=0.0098, Snap23KD: P=0.0508, Snap23KD+Rescue: P>0.9999
5b	αSyn Filament	Kruskal-Wallis	Dunn	0.0007	compared to control: Vamp7DN: P=0.0012, Snap23KD: P=0.0173, Snap23KD+Rescue: P>0.9999
5b	αSyn Amyloid	Kruskal-Wallis	Dunn	0.0026	compared to control: Vamp7DN: P=0.0061, Snap23KD: P=0.0121, Snap23KD+Rescue: P>0.9999
5b	Cathepsin-L	Kruskal-Wallis	Dunn	0.0013	compared to control: Vamp7DN: P=0.0029, Snap23KD: P=0.0115, Snap23KD+Rescue: P>0.9999
6a	K114: WT v. Tg^{x2}-αSyn^{A53T}	2-way ANOVA		**main effect culture media: F (1, 30) = 158.7, P<0.0001**; main effect incubation time: F (4, 30) = 81.43, P<0.0001; interaction: F (4, 30) = 16.60, P<0.0001	
6a	K114: Tg^{x2}-αSyn^{A53T} v. Tg^{x2}-αSyn^{A53T}+$Vamp7^{DN}$	2-way ANOVA		**main effect culture media: F (1, 30) = 15.24, P=0.0005**; main effect incubation time: F (4, 30) = 55.62, P<0.0001; interaction: F (4, 30) = 2.371, P=0.0747	
6b	Thioflavin T: WT v. Tg^{x2}-αSyn^{A53T}	2-way ANOVA		**main effect culture media: F (1, 30) = 77.84, P<0.0001**; main effect incubation time: F (4, 30) = 54.90, P<0.0001; interaction: F (4, 30) = 8.884, P<0.0001	
6b	Thioflavin T: Tg^{x2}-αSyn^{A53T} v. Tg^{x2}-αSyn^{A53T}+$Vamp7^{DN}$	2-way ANOVA		**main effect culture media: F (1, 30) = 4.627, P=0.0396**; main effect incubation time: F (4, 30) = 30.73, P<0.0001; interaction: F (4, 30) = 0.3030, P=0.8736	
6c	αSyn monomer: WT v. Tg^{x2}-αSyn^{A53T}	2-way ANOVA		**main effect culture media: F (1, 30) = 49.43, P<0.0001**; main effect incubation time: F (4, 30) = 50.19, P<0.0001; interaction: F (4, 30) = 6.521, P=0.0007	

6c	αSyn monomer: Tg^{x2}-$αSyn^{A53T}$ v. Tg^{x2}-$αSyn^{A53T}$+$Vamp7^{DN}$	2-way ANOVA		**main effect culture media: F (1, 30) = 18.29, P=0.0002**; main effect incubation time: F (4, 30) = 156.3, P<0.0001; interaction: F (4, 30) = 2.725, P=0.0478	
6c	αSyn Filament: WT v. Tg^{x2}-$αSyn^{A53T}$	2-way ANOVA		**main effect culture media: F (1, 30) = 72.83, P<0.0001**; main effect incubation time: F (4, 30) = 58.38, P<0.0001; interaction: F (4, 30) = 8.203, P=0.0001	
6c	αSyn Filament: Tg^{x2}-$αSyn^{A53T}$ v. Tg^{x2}-$αSyn^{A53T}$+$Vamp7^{DN}$	2-way ANOVA		**main effect culture media: F (1, 30) = 10.16, P=0.0033**; main effect incubation time: F (4, 30) = 51.19, P<0.0001; interaction: F (4, 30) = 1.262, P=0.3065	
6c	αSyn Amyloid: WT v. Tg^{x2}-$αSyn^{A53T}$	2-way ANOVA		**main effect culture media: F (1, 30) = 40.95, P<0.0001**; main effect incubation time: F (4, 30) = 53.45, P<0.0001; interaction: F (4, 30) = 8.326, P=0.0001	
6c	αSyn Amyloid: Tg^{x2}-$αSyn^{A53T}$ v. Tg^{x2}-$αSyn^{A53T}$+$Vamp7^{DN}$	2-way ANOVA		**main effect culture media: F (1, 30) = 6.193, P=0.0186**; main effect incubation time: F (4, 30) = 33.45, P<0.0001; interaction: F (4, 30) = 1.322, P=0.2843	
6d	αSyn monomer: WT v. Tg^{x2}-$αSyn^{A53T}$	2-way ANOVA		**main effect culture media: F (1, 16) = 3.153, P=0.0948**; main effect DIV: F (3, 16) = 1.257, P=0.3224; interaction: F (3, 16) = 0.9615, P=0.4349	
6d	αSyn monomer: Tg^{x2}-$αSyn^{A53T}$ v. Tg^{x2}-$αSyn^{A53T}$+$Vamp7^{DN}$	2-way ANOVA		**main effect culture media: F (1, 16) = 3.033, P=0.1008**; main effect DIV: F (3, 16) = 0.5580, P=0.6503; interaction: F (3, 16) = 0.2499, P=0.8602	
6d	αSyn Aggr.: WT v. Tg^{x2}-$αSyn^{A53T}$	2-way ANOVA		**main effect culture media: F (1, 16) = 64.17, P<0.0001**; main effect DIV: F (3, 16) = 106.7, P<0.0001; interaction: F (3, 16) = 11.02, P=0.0004	
6d	αSyn Aggr.: Tg^{x2}-$αSyn^{A53T}$ v. Tg^{x2}-$αSyn^{A53T}$+$Vamp7^{DN}$	2-way ANOVA		**main effect culture media: F (1, 16) = 10.88, P=0.0045**; main effect DIV: F (3, 16) = 113.7, P<0.0001; interaction: F (3, 16) = 4.776, P=0.0146	
6d	αSyn pSer129 monomer: WT v. Tg^{x2}-$αSyn^{A53T}$	2-way ANOVA		**main effect culture media: F (1, 16) = 1.101, P=0.3096**; main effect DIV: F (3, 16) = 2.589, P=0.0889; interaction: F (3, 16) = 0.5479, P=0.6567	
6d	αSyn pSer129 monomer: Tg^{x2}-$αSyn^{A53T}$ v. Tg^{x2}-$αSyn^{A53T}$+$Vamp7^{DN}$	2-way ANOVA		**main effect culture media: F (1, 16) = 1.081, P=0.3139**; main effect DIV: F (3, 16) = 0.3982, P=0.7561; interaction: F (3, 16) = 0.6218, P=0.6111	
6d	αSyn pSer129 Aggr.: WT v. Tg^{x2}-$αSyn^{A53T}$	2-way ANOVA		**main effect culture media: F (1, 16) = 112.0, P<0.0001**; main effect DIV: F (3, 16) = 172.2, P<0.0001; interaction: F (3, 16) = 23.23, P<0.0001	
6d	αSyn pSer129 Aggr.: Tg^{x2}-$αSyn^{A53T}$ v. Tg^{x2}-$αSyn^{A53T}$+$Vamp7^{DN}$	2-way ANOVA		**main effect culture media: F (1, 16) = 20.50, P=0.0003**; main effect DIV: F (3, 16) = 146.2, P<0.0001; interaction: F (3, 16) = 6.992, P=0.0032	
6d	αSyn Filament: WT v. Tg^{x2}-$αSyn^{A53T}$	2-way ANOVA		**main effect culture media: F (1, 16) = 31.17, P<0.0001**; main effect DIV: F (3, 16) = 133.7, P<0.0001; interaction: F (3, 16) = 4.206, P=0.0226	
6d	αSyn Filament: Tg^{x2}-$αSyn^{A53T}$ v. Tg^{x2}-$αSyn^{A53T}$+$Vamp7^{DN}$	2-way ANOVA		**main effect culture media: F (1, 16) = 4.040, P=0.0616**; main effect DIV: F (3, 16) = 136.2, P<0.0001; interaction: F (3, 16) = 0.8774, P=0.4735	
6d	αSyn Amyloid: WT v. Tg^{x2}-$αSyn^{A53T}$	2-way ANOVA		**main effect culture media: F (1, 16) = 154.8, P<0.0001**; main effect DIV: F (3, 16) = 514.3, P<0.0001; interaction: F (3, 16) = 52.81, P<0.0001	
6d	αSyn Amyloid: Tg^{x2}-$αSyn^{A53T}$ v. Tg^{x2}-$αSyn^{A53T}$+$Vamp7^{DN}$	2-way ANOVA		**main effect culture media: F (1, 16) = 49.51, P<0.0001**; main effect DIV: F (3, 16) = 541.5, P<0.0001; interaction: F (3, 16) = 13.17, P=0.0001	

S1	αSyn Monomer	2-way ANOVA	Bonferroni	**main effect genotype: F (1, 36) = 0.3040, P=0.5848**; main effect age: F (2, 36) = 0.09508, P=0.9095; interaction: F (2, 36) = 0.1884, P=0.8291	
S1	αSyn pSer129 Aggr.	2-way ANOVA	Bonferroni	**main effect genotype: F (1, 36) = 9.387, P=0.0041**; main effect age: F (2, 36) = 173.4, P<0.0001; interaction: F (2, 36) = 5.597, P=0.0076	comparison of genotype at each age: at 1 month: P>0.9999; at 3 month: P=0.0003 ; at 6 month: P=0.7434
S1	αSyn Filament	2-way ANOVA	Bonferroni	**main effect genotype: F (1, 36) = 21.44, P<0.0001**; main effect age: F (2, 36) = 162.4, P<0.0001; interaction: F (2, 36) = 2.516, P=0.0949	comparison of genotype at each age: at 1 month: P=0.8570; at 3 month: P=0.0004; at 6 month: P=0.0331
S1	αSyn Amyloid	2-way ANOVA	Bonferroni	**main effect genotype: F (1, 36) = 18.44, P=0.0001**; main effect age: F (2, 36) = 196.4, P<0.0001; interaction: F (2, 36) = 3.185, P=0.0532	comparison of genotype at each age: at 1 month: P>0.9999; at 3 month: P=0.0005; at 6 month: P=0.0365
S1	LAMP1	2-way ANOVA	Bonferroni	**main effect genotype: F (1, 36) = 74.59, P<0.0001**; main effect age: F (2, 36) = 15.37, P<0.0001; interaction: F (2, 36) = 22.74, P<0.0001	comparison of genotype at each age: at 1 month: P>0.9999; at 3 month: P<0.0001; at 6 month: P<0.0001
S1	Cathepsin-L	2-way ANOVA	Bonferroni	**main effect genotype: F (1, 36) = 101.9, P<0.0001**; main effect age: F (2, 36) = 31.75, P<0.0001; interaction: F (2, 36) = 38.88, P<0.0001	comparison of genotype at each age: at 1 month: P>0.9999; at 3 month: P<0.0001; at 6 month: P<0.0001
S1	ATP5G	2-way ANOVA	Bonferroni	**main effect genotype: F (1, 36) = 51.42, P<0.0001**; main effect age: F (2, 36) = 22.94, P<0.0001; interaction: F (2, 36) = 20.54, P<0.0001	comparison of genotype at each age: at 1 month: P>0.9999; at 3 month: P=0.0045; at 6 month: P<0.0001
S1	NeuN	2-way ANOVA	Bonferroni	**main effect genotype: F (1, 36) = 0.8585, P=0.3603**; main effect age: F (2, 36) = 0.9601, P=0.3925; interaction: F (2, 36) = 0.6756, P=0.5152	
S3b		RM 1-way ANOVA (matched by litter)		**genotype: F (3, 24) = 0.004286, P=0.9996**; mouse: F (8, 24) = 6.399e-006, P>0.9999	
S3f	WT v. Tg- ^{HA}Lamp1^{Myc}	RM 2-way ANOVA (each mouse matched over age)		**main effect genotype: F (1, 10) = 0.002403, P=0.9619**; main effect age: F (21, 210) = 3.080, P<0.0001; interaction: F (21, 210) = 0.2269, P=0.9999; mouse: F (10, 210) = 2.201, P=0.0189	
S3f	Tg-αSyn^{A53T} v. Tg-αSyn^{A53T}/Tg-^{HA}Lamp1^{Myc} (with 20s placeholder, darker curves)	RM 2-way ANOVA (each mouse matched over age)		**main effect genotype: F (1, 18) = 0.0002800, P=0.9868**; main effect age: F (21, 378) = 39.55, P<0.0001; interaction: F (21, 378) = 0.1851, P>0.9999; mouse: F (18, 378) = 20.62, P<0.0001	
S3f	Tg-αSyn^{A53T} v. Tg-αSyn^{A53T}/Tg-^{HA}Lamp1^{Myc} (lighter curves)	mixed effects analysis(each mouse matched over age)		**main effect genotype: F (1, 18) = 5.690e-006, P=0.9981**; main effect age: F (21, 237) = 9.570, P<0.0001	
S3g	WT v. Tg- ^{HA}Lamp1^{Myc}	RM 2-way ANOVA (each mouse matched over age)		**main effect genotype: F (1, 10) = 2.241, P=0.1653**; main effect age: F (21, 210) = 0.6585, P=0.8701; interaction: F (21, 210) = 0.8670, P=0.6340; mouse: F (10, 210) = 1.293, P=0.2362	

S3g	Tg-αSyn^{A53T} v. Tg-αSyn^{A53T}/Tg-^{HA}Lamp1^{Myc} (with 20s placeholder, darker curves)	RM 2-way ANOVA (each mouse matched over age)		**main effect genotype: F (1, 18) = 0.001361, P=0.9710**; main effect age: F (21, 378) = 40.67, P<0.0001; interaction: F (21, 378) = 0.3031, P=0.9990; mouse: F (18, 378) = 19.32, P<0.0001	
S3g	Tg-αSyn^{A53T} v. Tg-αSyn^{A53T}/Tg-^{HA}Lamp1^{Myc} (lighter curves)	mixed effects analysis(each mouse matched over age)		**main effect genotype: F (1, 18) = 0.1290, P=0.7237**; main effect age: F (21, 237) = 8.908, P<0.0001	
S4d	Tg^{x2}-αSyn^{A53T} v. Tg-αSyn^{A53T} (with 20s placeholder, darker curves) up 182 days (~6mo)	RM 2-way ANOVA (each mouse matched over age)		**main effect genotype: F (1, 21) = 20.14, P=0.0002**; main effect age: F (11, 231) = 32.18, P<0.0001; interaction: F (11, 231) = 13.11, P<0.0001; mouse: F (21, 231) = 8.424, P<0.0001	
S4d	Tg-αSyn^{A53T} v. WT (with 20s placeholder, darker curves)	RM 2-way ANOVA (each mouse matched over age)		**main effect genotype: F (1, 14) = 13.52, P=0.0025**; main effect age: F (22, 308) = 8.178, P<0.0001; interaction: F (22, 308) = 9.294, P<0.0001; mouse: F (14, 308) = 18.24, P<0.0001	
S4d	Tg^{x2}-αSyn^{A53T} v. Tg-αSyn^{A53T} (lighter curves) up 182 days (~6mo)	mixed effects analysis(each mouse matched over age)		**main effect genotype: F (1, 21) = 39.48, P<0.0001**; main effect age: F (11, 178) = 15.77, P<0.0001; interaction: F (11, 178) = 9.693, P<0.0001	
S4d	Tg-αSyn^{A53T} v. WT (lighter curves)	mixed effects analysis(each mouse matched over age)		**main effect genotype: F (1, 14) = 25.50, P=0.0002**; main effect age: F (22, 246) = 4.851, P<0.0001; interaction: F (22, 246) = 5.510, P<0.0001	
S4e	Tg^{x2}-αSyn^{A53T} v. Tg-αSyn^{A53T} (with 20s placeholder, darker curves) up 182 days (~6mo)	RM 2-way ANOVA (each mouse matched over age)		**main effect genotype: F (1, 21) = 21.05, P=0.0002**; main effect age: F (11, 231) = 31.95, P<0.0001; interaction: F (11, 231) = 11.70, P<0.0001; mouse: F (21, 231) = 7.653, P<0.0001	
S4e	Tg-αSyn^{A53T} v. WT (with 20s placeholder, darker curves)	RM 2-way ANOVA (each mouse matched over age)		**main effect genotype: F (1, 14) = 13.86, P=0.0023**; main effect age: F (22, 308) = 9.009, P<0.0001; interaction: F (22, 308) = 8.546, P<0.0001; mouse: F (14, 308) = 15.24, P<0.0001	
S4e	Tg^{x2}-αSyn^{A53T} v. Tg-αSyn^{A53T} (lighter curves) up 182 days (~6mo)	mixed effects analysis(each mouse matched over age)		**main effect genotype: F (1, 21) = 42.95, P<0.0001**; main effect age: F (11, 178) = 12.74, P<0.0001; interaction: F (11, 178) = 6.847, P<0.0001	
S4e	Tg-αSyn^{A53T} v. WT (lighter curves)	mixed effects analysis(each mouse matched over age)		**main effect genotype: F (1, 14) = 35.45, P<0.0001**; main effect age: F (22, 246) = 5.189, P<0.0001; interaction: F (22, 246) = 4.551, P<0.0001	
S4f	αSyn Aggr.	2-way ANOVA		**main effect genotype: F (1, 30) = 608.0, P<0.0001**; main effect age: F (4, 30) = 236.2, P<0.0001; interaction: F (4, 30) = 242.2, P<0.0001	
S4f	αSyn Filament	2-way ANOVA		**main effect genotype: F (1, 30) = 398.1, P<0.0001**; main effect age: F (4, 30) = 167.2, P<0.0001; interaction: F (4, 30) = 172.8, P<0.0001	
S4f	αSyn Amyloid	2-way ANOVA		**main effect genotype: F (1, 30) = 638.4, P<0.0001**; main effect age: F (4, 30) = 359.1, P<0.0001; interaction: F (4, 30) = 345.4, P<0.0001	
S4f	αSyn Aggr. at day 56	1-way ANOVA	Bonferroni	F (2, 9) = 184.3, P<0.0001	WT vs. Tg-aSynA53T: P>0.9999, WT vs. 2xTg-aSynA53T: P<0.0001, Tg-aSynA53T vs. 2xTg-aSynA53T: P<0.0001
S4f	αSyn Filament at day 56	1-way ANOVA	Bonferroni	F (2, 9) = 677.0, P<0.0001	WT vs. Tg-aSynA53T: P>0.9999, WT vs. 2xTg-aSynA53T: P<0.0001, Tg-aSynA53T vs. 2xTg-aSynA53T: P<0.0001
S4f	αSyn Amyloid at day 56	1-way ANOVA	Bonferroni	F (2, 9) = 728.0, P<0.0001	WT vs. Tg-aSynA53T: P>0.9999, WT vs. 2xTg-aSynA53T: P<0.0001, Tg-

					aSynA53T vs. 2xTg-aSynA53T: P<0.0001
S5a	αSyn Monomer	2-way ANOVA		**main effect genotype: F (1, 30) = 1030, P<0.0001**; main effect age: F (4, 30) = 0.1917, P=0.9409; interaction: F (4, 30) = 0.2992, P=0.8761	
S5a	αSyn Aggr.	2-way ANOVA		**main effect genotype: F (1, 30) = 209.3, P<0.0001**; main effect age: F (4, 30) = 103.7, P<0.0001; interaction: F (4, 30) = 110.3, P<0.0001	
S5a	αSyn Filament	2-way ANOVA		**main effect genotype: F (1, 30) = 635.7, P<0.0001**; main effect age: F (4, 30) = 417.1, P<0.0001; interaction: F (4, 30) = 406.8, P<0.0001	
S5a	αSyn Amyloid	2-way ANOVA		**main effect genotype: F (1, 30) = 295.5, P<0.0001**; main effect age: F (4, 30) = 205.1, P<0.0001; interaction: F (4, 30) = 209.3, P<0.0001	
S5e		1-way ANOVA	Dunnett	F (2, 6) = 0.5843, P=0.5864	compared to DIV35: at DIV42: P=0.5298; at DIV49: P=0.6402
S5f		1-way ANOVA	Dunnett	F (2, 9) = 0.2666, P=0.7718	compared to DIV35: at DIV42: P=0.8868; at DIV49: P=0.6980
S6	Tg^{x2}-αSyn^{A53T} DIV49	1-way ANOVA	Dunnett	F (5, 24) = 12.97, P<0.0001	compared to monomer: Aggr: P=0.0014, pSer129 mono: P>0.9999, pSer129 Aggr: P=0.0068, Fila: P=0.0001, Amyl: P<0.0001
S6	Tg^{x2}-αSyn^{A53T} DIV49	Kruskal-Wallis	Dunn	0.0006	compared to monomer: Aggr: P=0.0353, pSer129 mono: P>0.9999, pSer129 Aggr: P=0.2212, Fila: P=0.0079, Amyl: P=0.0032
S8a	αSyn Aggr.	1-way ANOVA	Dunnett	F (4, 20) = 25.56, P<0.0001	compared to vehicle: TTX: P=0.0805, CNQX/APV: P=0.0322, K+: P=0.0004, Bicuculline: P=0.0023
S8a	αSyn pSer129 Aggr.	1-way ANOVA	Dunnett	F (4, 20) = 38.42, P<0.0001	compared to vehicle: TTX: P=0.0031, CNQX/APV: P=0.0002, K+: P=0.0013, Bicuculline: P=0.0016
S8a	αSyn Filament	1-way ANOVA	Dunnett	F (4, 20) = 42.33, P<0.0001	compared to vehicle: TTX: P=0.0052, CNQX/APV: P=0.0001, K+: P=0.0002, Bicuculline: P=0.0032
S8a	αSyn Amyloid	1-way ANOVA	Dunnett	F (4, 20) = 63.01, P<0.0001	compared to vehicle: TTX: P=0.0004, CNQX/APV: P<0.0001, K+: P<0.0001, Bicuculline: P<0.0001
S8a	Cathepsin-L	1-way ANOVA	Dunnett	F (4, 20) = 85.75, P<0.0001	compared to vehicle: TTX: P=0.0003, CNQX/APV: P<0.0001, K+: P<0.0001, Bicuculline: P<0.0001
S8b	αSyn Aggr.	1-way ANOVA	Dunnett	F (2, 12) = 13.06, P=0.0010	compared to vehicle: Dan/2-APB: P=0.0008, YM-58483: P=0.0043
S8b	αSyn pSer129 Aggr.	1-way ANOVA	Dunnett	F (2, 12) = 24.24, P<0.0001	compared to vehicle: Dan/2-APB: P<0.0001, YM-58483: P=0.0009

S8b	αSyn Filament	1-way ANOVA	Dunnett	F (2, 12) = 29.60, P<0.0001	compared to vehicle: Dan/2-APB: P<0.0001, YM-58483: P=0.0004
S8b	αSyn Amyloid	1-way ANOVA	Dunnett	F (2, 12) = 64.91, P<0.0001	compared to vehicle: Dan/2-APB: P<0.0001, YM-58483: P<0.0001
S8b	Cathepsin-L	1-way ANOVA	Dunnett	F (2, 12) = 16.52, P=0.0004	compared to vehicle: Dan/2-APB: P=0.0003, YM-58483: P=0.0028
S8b	αSyn Aggr.	Kruskal-Wallis	Dunn	0.001	compared to vehicle: Dan/2-APB: P=0.0055, YM-58483: P=0.0313
S8b	αSyn pSer129 Aggr.	Kruskal-Wallis	Dunn	0.0003	compared to vehicle: Dan/2-APB: P=0.0024, YM-58483: P=0.0615
S8b	αSyn Filament	Kruskal-Wallis	Dunn	0.0005	compared to vehicle: Dan/2-APB: P=0.0031, YM-58483: P=0.0512
S8b	αSyn Amyloid	Kruskal-Wallis	Dunn	<0.0001	compared to vehicle: Dan/2-APB: P=0.0014, YM-58483: P=0.0876
S8b	Cathepsin-L	Kruskal-Wallis	Dunn	0.0007	compared to vehicle: Dan/2-APB: P=0.0039, YM-58483: P=0.0424
S8c	Neuronal activity	1-way ANOVA	Dunnett	F (4, 20) = 0.1921, P=0.9397	compared to vehicle: TTX: P=0.9403, CNQX/APV: P=0.9482, K+: P=0.9678, Bicuculline: P>0.9999
S8c	Calcium inhibition	1-way ANOVA	Dunnett	F (2, 12) = 0.1453, P=0.8663	compared to vehicle: Dan/2-APB: P=0.8331, YM-58483: P=0.8859
S8d		1-way ANOVA	Dunnett	F (6, 14) = 0.8795, P=0.5344	compared to vehicle: TTX: P=0.937, CNQX/APV: P=0.9, K+: P, Bicuculline: P=0.7639, Dan/2-APB: P=0.9588, YM-58483: P=0.9982
S9b	VAMP7	1-way ANOVA	Dunnett	F (2, 9) = 14.84, P=0.0014	compared to control: Vamp7KD: P=0.0019, Snap23KD: P=0.9948
S9b	SNAP23	1-way ANOVA	Dunnett	F (2, 9) = 82.85, P<0.0001	compared to control: Vamp7KD: P=0.7031, Snap23KD: P<0.0001
S9b	Cathepsin-L	1-way ANOVA	Dunnett	F (5, 18) = 30.56, P<0.0001	compared to WT control: WT Vamp7KD: P=0.9996, WT Snap23KD: P<0.0001, Tg^{x2}-αSyn^{A53T} control: P=0.9976, Tg^{x2}-αSyn^{A53T} Vamp7KD: P=0.9997, Tg^{x2}-αSyn^{A53T} Snap23KD: P<0.0001
S9b	Neuroserpin	1-way ANOVA	Dunnett	F (5, 18) = 0.6526, P=0.6634	
S9b	VAMP7	Kruskal-Wallis	Dunn	0.0026	compared to control: Vamp7KD: P=0.0104, Snap23KD: P=0.8491
S9b	SNAP23	Kruskal-Wallis	Dunn	0.0026	compared to control: Vamp7KD: P=0.8491, Snap23KD: P=0.0104
S9b	Cathepsin-L	Kruskal-Wallis	Dunn	0.0067	compared to WT control: WT Vamp7KD: P>0.9999, WT Snap23KD: P=0.0695, Tg^{x2}-αSyn^{A53T} control: P>0.9999, Tg^{x2}-αSyn^{A53T} Vamp7KD: P>0.9999, Tg^{x2}-αSyn^{A53T} Snap23KD: P=0.0916
S9b	Neuroserpin	Kruskal-Wallis	Dunn	0.6459	
S9c	Cathepsin-L	1-way ANOVA	Dunnett	F (5, 18) = 165.7, P<0.0001	compared to WT control: WT Vamp7DN: P<0.0001,

					WT Snap23DN: P=0.3205, Tg^{x2}-αSyn^{A53T} control: P>0.9999, Tg^{x2}-αSyn^{A53T} Vamp7DN: P<0.0001, Tg^{x2}-αSyn^{A53T} Snap23DN: P=0.3804
S9c	Neuroserpin	1-way ANOVA	Dunnett	F (5, 18) = 0.2564, P=0.9310	
S9c	Cathepsin-L	Kruskal-Wallis	Dunn	0.0034	compared to WT control: WT Vamp7DN: P=0.0155, WT Snap23DN: P>0.9999, Tg^{x2}-αSyn^{A53T} control: P>0.9999, Tg^{x2}-αSyn^{A53T} Vamp7DN: P=0.0111, Tg^{x2}-αSyn^{A53T} Snap23DN: P>0.9999
S9c	Neuroserpin	Kruskal-Wallis	Dunn	0.9043	
S9d		1-way ANOVA	Dunnett	F (2, 6) = 0.2295, P=0.8016	compared to control: Vamp7DN: P=0.9035, Snap23KD: P=0.9377
S10b	αSyn Monomer	2-way ANOVA	Bonferroni	**main effect culture media: F (3, 36) = 0.01230, P=0.9981**; main effect incubation time: F (2, 36) = 816.5, P<0.0001; interaction: F (6, 36) = 0.003696, P>0.9999	compared to no media: unconditioned media: P>0.9999, αSyn-/- media: P>0.9999, WT media: P>0.9999
S10b	αSyn Filament	2-way ANOVA	Bonferroni	**main effect culture media: F (3, 36) = 0.2541, P=0.8579**; main effect incubation time: F (2, 36) = 3059, P<0.0001; interaction: F (6, 36) = 0.09652, P=0.9963	compared to no media: unconditioned media: P>0.9999, αSyn-/- media: P>0.9999, WT media: P>0.9999
S10b	αSyn Amyloid	2-way ANOVA	Bonferroni	**main effect culture media: F (3, 36) = 0.4170, P=0.7418**; main effect incubation time: F (2, 36) = 1995, P<0.0001; interaction: F (6, 36) = 0.1400, P=0.9899	compared to no media: unconditioned media: P>0.9999, αSyn-/- media: P=0.8530, WT media: P>0.9999

SUPPLEMENTARY REFERENCES

1 Abu-Remaileh, M. *et al.* Lysosomal metabolomics reveals V-ATPase- and mTOR-dependent regulation of amino acid efflux from lysosomes. *Science* **358**, 807-813, doi:10.1126/science.aan6298 (2017).

2 Wyant, G. A. *et al.* NUFIP1 is a ribosome receptor for starvation-induced ribophagy. *Science* **360**, 751-758, doi:10.1126/science.aar2663 (2018).
