# Supplementary material for: Lysosomal exocytosis releases pathogenic α-synuclein species from neurons in synucleinopathy models
Source: Nat Commun. 2022 Aug 22;13:4918. doi: 10.1038/s41467-022-32625-1 (PMC9395532; doi:10.1038/s41467-022-32625-1)
Supplement: Supplementary file 3 — Reporting Summary [file 41467_2022_32625_MOESM3_ESM.pdf]

nature research

Corresponding author(s): Manu Sharma

Last updated by author(s): Jul 21, 2022

Reporting Summary

Nature Research wishes to improve the reproducibility of the work that we publish. This form provides structure for consistency and transparency in reporting. For further information on Nature Research policies, see our Editorial Policies and the Editorial Policy Checklist.

Statistics

For all statistical analyses, confirm that the following items are present in the figure legend, table legend, main text, or Methods section.

n/a	Confirmed	
☐	☒	The exact sample size (n) for each experimental group/condition, given as a discrete number and unit of measurement
☐	☒	A statement on whether measurements were taken from distinct samples or whether the same sample was measured repeatedly
☐	☒	The statistical test(s) used AND whether they are one- or two-sided<br>*Only common tests should be described solely by name; describe more complex techniques in the Methods section.*
☐	☒	A description of all covariates tested
☐	☒	A description of any assumptions or corrections, such as tests of normality and adjustment for multiple comparisons
☐	☒	A full description of the statistical parameters including central tendency (e.g. means) or other basic estimates (e.g. regression coefficient) AND variation (e.g. standard deviation) or associated estimates of uncertainty (e.g. confidence intervals)
☐	☒	For null hypothesis testing, the test statistic (e.g. F, t, r) with confidence intervals, effect sizes, degrees of freedom and P value noted<br>*Give P values as exact values whenever suitable.*
☒	☐	For Bayesian analysis, information on the choice of priors and Markov chain Monte Carlo settings
☒	☐	For hierarchical and complex designs, identification of the appropriate level for tests and full reporting of outcomes
☒	☐	Estimates of effect sizes (e.g. Cohen's d, Pearson's r), indicating how they were calculated

Our web collection on statistics for biologists contains articles on many of the points above.

Software and code

Policy information about availability of computer code

Data collection	No computer code was written specifically for this study. See "Methods" for commercial equipment and software used to collect data.
Data analysis	No computer code was written specifically for this study. See "Methods" section for software used to analyze data.

For manuscripts utilizing custom algorithms or software that are central to the research but not yet described in published literature, software must be made available to editors and reviewers. We strongly encourage code deposition in a community repository (e.g. GitHub). See the Nature Research guidelines for submitting code & software for further information.

Data

Policy information about availability of data

All manuscripts must include a data availability statement. This statement should provide the following information, where applicable:

- Accession codes, unique identifiers, or web links for publicly available datasets
- A list of figures that have associated raw data
- A description of any restrictions on data availability

No restrictions on data availability. The data that support the findings in this study are available, firstly in Source Data files, and any others from the corresponding author upon reasonable request.

Field-specific reporting

For a reference copy of the document with all sections, see nature.com/documents/nr-reporting-summary-flat.pdf

Life sciences study design

All studies must disclose on these points even when the disclosure is negative.

Sample size	Figure legends and the Methods "Statistical analyses" subsection.
Data exclusions	No data excluded. Also, see Methods, "Statistical analyses" subsection.
Replication	Independent replicants of experiments are included as separate "n"s, with no data excluded. Also, see Methods, "Statistical analyses" subsection.
Randomization	Methods, "Statistical analyses" subsection.
Blinding	Methods, "Statistical analyses" subsection.

Reporting for specific materials, systems and methods

We require information from authors about some types of materials, experimental systems and methods used in many studies. Here, indicate whether each material, system or method listed is relevant to your study. If you are not sure if a list item applies to your research, read the appropriate section before selecting a response.

Materials & experimental systems

n/a	Involved in the study	
☐	☒	Antibodies
☐	☒	Eukaryotic cell lines
☒	☐	Palaeontology and archaeology
☐	☒	Animals and other organisms
☒	☐	Human research participants
☒	☐	Clinical data
☒	☐	Dual use research of concern

Methods

n/a	Involved in the study	
☒	☐	ChIP-seq
☒	☐	Flow cytometry
☒	☐	MRI-based neuroimaging

Antibodies

Antibodies used	See Methods "Antibody list" subsection for all the antibodies used and their sources.
Validation	Primary antibodies are validated by the source companies (listed in Methods section), previous use in unrelated published studies, as well as confirmed by us via recognition of a correctly-sized band on immunoblots.

Eukaryotic cell lines

Policy information about cell lines

Cell line source(s)	See Methods for the eukaryotic cell lines used for the study.
Authentication	Cell lines were purchased from ATCC, and thawed every 15 days to keep a low passage number. Also, see Methods.
Mycoplasma contamination	Mycoplasma testing in all the cell lines used here was negative. Also, see Methods.
Commonly misidentified lines (See ICLAC register)	Only cell line used Hek293T, was purchased directly from ATCC. Also, see Methods.

Animals and other organisms

Policy information about studies involving animals; ARRIVE guidelines recommended for reporting animal research

Laboratory animals	See Methods section for mouse strains used, and the institutional boards that approved the animal protocols.
Wild animals	N/A
Field-collected samples	N/A
Ethics oversight	Institutional Animal Care and Use Committee (IACUC) at Weill Cornell Medicine and NIH.

Note that full information on the approval of the study protocol must also be provided in the manuscript.
